# Complete genome sequence of *Lactobacillus rhamnosus* Pen, a probiotic component of a medicine used in prevention of antibiotic-associated diarrhoea in children

**DOI:** 10.1186/s13099-018-0235-z

**Published:** 2018-02-22

**Authors:** Piotr Jarocki, Marcin Podleśny, Mariusz Krawczyk, Agnieszka Glibowska, Jarosław Pawelec, Elwira Komoń-Janczara, Oleksandr Kholiavskyi, Michał Dworniczak, Zdzisław Targoński

**Affiliations:** 1 Department of Biotechnology, Microbiology and Human Nutrition, University of Life Sciences in Lublin, 8 Skromna St., 20-704 Lublin, Poland; 2 Process and Development Department, Grupa Azoty Zakłady Azotowe “Puławy” S.A, Al. Tysiąclecia Państwa Polskiego 13, 24-110 Puławy, Poland; 3 Genomed SA, Ponczowa 12, 02-971 Warsaw, Poland; 4 Laboratory of Electron Microscopy, Department of Comparative Anatomy and Anthropology, Maria Curie-Skłodowska University, 19 Akademicka St., 20-033 Lublin, Poland

**Keywords:** *Lactobacillus rhamnosus* Pen, Probiotics, Genome sequence, CRISPR–Cas locus, Prophage

## Abstract

**Background:**

*Lactobacillus rhamnosus* Pen is a human endogenous strain with well-documented health promoting properties that is used for production of probiotics. It has a long safety history of application, and its effectiveness in the prevention of antibiotic-associated diarrhoea has also been confirmed in clinical trials.

**Results:**

Here we present the complete genome sequence of *L. rhamnosus* Pen, which consists of a circular 2,884,4966-bp chromosome with a GC content of 46.8%. Within 2907 open reading frames (ORFs), genes involved with probiotic properties were identified. A CRISPR locus, consisting of a 1092-nt region with 16 spacers, was also detected. Finally, an intact prophage of ~ 40.7 kb, 57 ORFs, GC content 44.8% was identified.

**Conclusions:**

Genomic analysis confirmed the probiotic properties of *L. rhamnosus* Pen and may indicate new biotechnological applications of this industrially important strain.

**Electronic supplementary material:**

The online version of this article (10.1186/s13099-018-0235-z) contains supplementary material, which is available to authorized users.

## Introduction

*Lactobacillus rhamnosus* has been isolated from the human intestinal tract, oral cavity, and vagina. Owing to their beneficial effects on human health, many strains of *L. rhamnosus* are also used in the dairy and pharmaceutical industries. Examples of such industrially important probiotic strains are *Lactobacillus rhamnosus* GG and *Lactobacillus rhamnosus* R0011, as well as *Lactobacillus rhamnosus* Pen, which is a component of a medicine commonly used to reduce the risk of diarrhoea development during antibiotic therapy [1–3]. Many characteristics of strain Pen have previously been reported, including carbohydrate utilisation, colony and cell morphology, antibiotic sensitivity, RAPD patterns, and SDS-PAGE and two-dimensional (2D) electrophoretic profiles of surface-associated proteins [4, 5]. Other properties, such as adhesion ability [6], survival rate in acidic pH [7], antiradical activity [8] and production of extracellular ferulic acid esterase [9] have also been analysed. Optimisation of medium composition to enhance growth of *L. rhamnosus* Pen using response surface methodology was reported by Polak-Berecka et al. [10].

## Methods

Genomic DNA was isolated and purified using a Genomic Mini AX Bacteria + kit (A&A Biotechnology, Gdynia, Poland); DNA concentration was determined using a NanoDrop spectrophotometer (Thermo Scientific, Waltham, USA). Sequencing was performed at Genomed SA. Briefly, a paired-end library was constructed by using the NEB-Next^®^ DNA Library Prep Master Mix Set for Illumina (NEB, Ipswich, USA) and subsequently sequenced on an Illumina MiSeq with 2 × 250 paired end sequencing chemistry (Illumina, San Diego, USA). Additionally, a 5–8 kb mate-pair library was constructed according protocol developed in BGI (Shenzhen, China) and sequenced on a HiSeq 4000 with 2 × 100 paired end sequencing chemistry (Illumina, San Diego, USA). A total of 1,270,358,608 bases and 362,759,422 paired reads were yielded. Read trimming and filtering was performed using Cutadapt 1.9.1 [11]. De novo assembly was conducted using SPAdes 3.1.1. [12], which yielded one major contig with 679-fold average coverage. Functional annotation of predicted genes was performed using the NCBI Prokaryotic Genome Annotation Pipeline [13]. The clusters of orthologous groups (COGs) of proteins were determined using eggNOG 4.5 [14]. Ribosomal RNA genes were detected using RNAmer 1.2 [15] and tRNA genes were identified using tRNAscan-SE v. 2.0 [16]. Sequences of proteins which may determine putative probiotic properties of *L. rhamnosus* Pen were individually search against Conserved Domains Database (NCBI) [17] and InterPro detabase (EMBL-EBI) [18]. Genes potentially involved in the biosynthesis of bacteriocins were identified using BAGEL [19]. The presence of antibiotic resistance genes was tested using ResFinder [20]. Phaster was used to search for prophage sequences [21] and the presence of a CRISPR/Cas system was predicted using CRISPRs finder [22] and the Crispr Recognition Tool [23]. Genome mapping and alignment visualisation were performed using CGView [24] and BRIG [25] respectively.

### Quality assurance

Genomic DNA used for sequencing was isolated from a pure culture of a single bacterial isolate of *Lactobacillus rhamnosus* Pen (Additional file 1: Figure S1). Additionally, the 16S rRNA gene sequence was determined and compared against NCBI database using BLAST (Additional file 2: Figure S2).

## Results and discussion

**Fig. 1:**
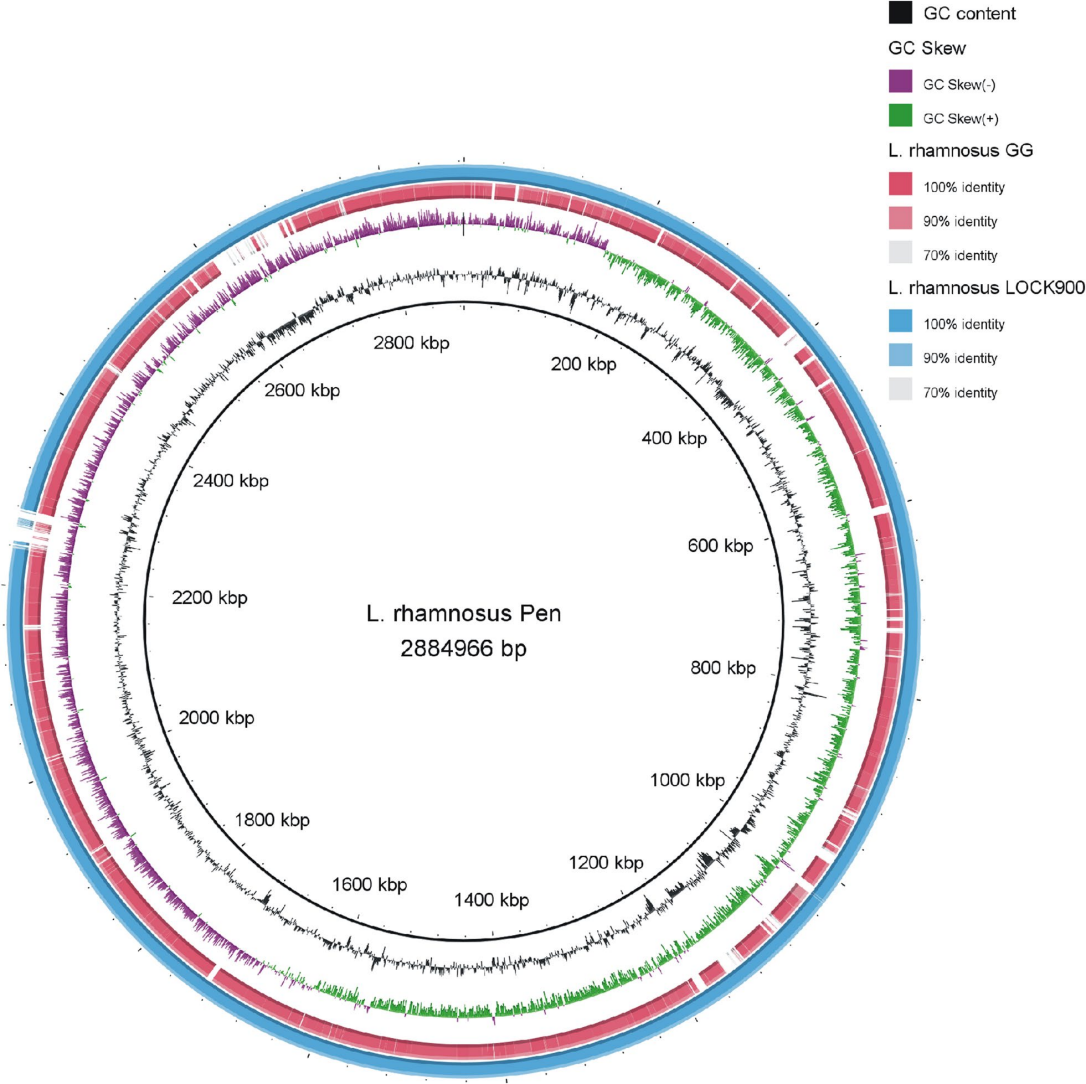
Visualization of alignment of the *Lactobacillus rhamnosus* Pen genome with *Lactobacillus rhamnosus* LOCK900 and *Lactobacillus rhamnosus* GG complete genome sequences

The complete genome of *L. rhamnosus* Pen consists of a 2,884,966-nt circular chromosome (GC content of 46.8%) with no plasmid. Among the 2907 identified open reading frames, 2729 contain protein-coding genes. In addition, 59 tRNA genes, 5 rRNA operons, and 101 pseudogenes were identified (Table 1, Additional file 3: Figure S3). Of the identified coding sequences, 2422 (88.7%) were grouped into 20 COG classes. Coding sequences were identified as being involved in carbohydrate transport and metabolism (12%), transcription (7.3%), amino acid transport and metabolism (6.9%), translation, ribosomal structure and biogenesis (5.4%), and replication, recombination and repair of nucleic acids (4.8%) (Table 2, Additional file 3: Figure S3). Comparison of the *L. rhamnosus* Pen genome with eleven other *L. rhamnosus* complete genome sequences showed the highest similarity with intestinal isolate *L. rhamnosus* LOCK900 (symmetric identity 98.76%, gapped identity 99.97; CP005484.1) [26] and substantially lower sequence similarity with the industrially important *L. rhamnosus* GG (symmetric identity 84.24%, gapped identity 97.50%; AP011548.1) [27] (Fig. 1).

**Table 1:** General features of *Lactobacillus rhamnosus* Pen genome

Attribute	Value
Genome size (bp)	2,884,966
Contig numbers	1
DNA G+C (%)	46.8
Total genes	2907
Protein-coding genes	2729
rRNA genes	15
tRNA genes	59
ncRNA genes	3
Pseudogenes	101
Plasmid	0
Prophages	1
CRISPR arrays	1
GenBank accession	CP020464.1

**Table 2:** COG functional categories of *Lactobacillus rhamnosus* Pen genome

#COG class	Description	Count	%
Information storage and processing			
[J]	Translation, ribosomal structure and biogenesis	153	5.4
[A]	RNA processing and modification	0	0.0
[K]	Transcription	208	7.3
[L]	Replication, recombination and repair	135	4.8
[B]	Chromatin structure and dynamics	0	0.0
Cellular processes and signaling			
[D]	Cell cycle control, cell division, chromosome partitioning	34	1.2
[Y]	Nuclear structure	0	0.0
[V]	Defense mechanisms	101	3.6
[T]	Signal transduction mechanisms	97	3.4
[M]	Cell wall/membrane/envelope biogenesis	130	4.6
[N]	Cell motility	9	0.3
[Z]	Cytoskeleton	0	0.0
[W]	Extracellular structures	0	0.0
[U]	Intracellular trafficking, secretion, and vesicular transport	23	0.8
[O]	Posttranslational modification, protein turnover, chaperones	57	2.0
Metabolism			
[C]	Energy production and conversion	91	3.2
[G]	Carbohydrate transport and metabolism	339	12.0
[E]	Amino acid transport and metabolism	195	6.9
[F]	Nucleotide transport and metabolism	87	3.1
[H]	Coenzyme transport and metabolism	57	2.0
[I]	Lipid transport and metabolism	62	2.2
[P]	Inorganic ion transport and metabolism	103	3.6
[Q]	Secondary metabolites biosynthesis, transport and catabolism	27	1.0
Poorly characterized			
[R]	General function prediction only	303	10.7
[S]	Function unknown	211	7.5

Comparative genomic analysis of *L. rhamnosus* Pen showed the presence of numerous genes which may determine its putative probiotic properties, supporting use of the strain in prevention of various gastrointestinal disorders. Genetic factors involved in cell surface adherence, biofilm formation, and pathogen inhibition were identified (Additional file 4: Table S1). Such features are known to provide a survival advantage for probiotic strains and are important for effective bacterial colonisation of the human intestine [1, 28–32]. Additionally, detailed analysis of the genome did not reveal transmissible antibiotic resistance genes in the chromosome of *L*. *rhamnosus* Pen. It was previously described that such genetic determinants may constitute a reservoir of antibiotic resistance for food and gut pathogens. On the other hand, presence of intrinsic antibiotic resistance among probiotic strains is valuable factor in restoring the intestinal microbiota after antibiotic treatment [33].

**Fig. 2:**
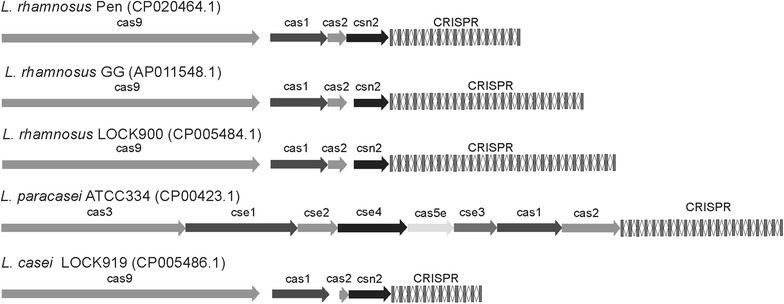
CRISPR–Cas system architecture of selected *Lactobacillus rhamnosus* strains

The analysis performed using CRISPRs finder and the Crispr Recognition Tool indicated that the genome contains one regularly interspaced short palindromic repeat locus consisting of a 1092-nt region with 16 spacers (30–31 nt in length) (Fig. 2). The detected CRISPR–Cas system is of type II-A/LsaI1 (four cas genes; cas1, cas2, cas9, csn2, and one CRISPR array), similar to previously described CRISPR loci characteristic of *L. rhamnosus* strains [34]. BLASTN searches comparing all 16 spacers against the phage and plasmid NCBI databases revealed no sequence identity with known mobile genetic elements of lactobacilli. In a previous report, Douillard et al. [29] observed that many spacer sequences of *L. rhamnosus* strains fully or partially matched sequenced bacteriophage genomes, such as *Lactobacillus rhamnosus* phage Lc-Nu and Lrm1, as well as *L. casei* phages, including φAT3, A2, and PL-1. This phenomenon suggests that CRISPR modules may play an important role in protection against different mobile elements and also provide specific bacteriophage resistance [35]. Interestingly, similar results were not obtained for the CRISPR locus identified for *Lactobacillus rhamnosus* Pen.

Finally, one intact prophage of ~ 40.7 kb with a GC content of 44.8% was identified. This prophage sequence showed only 94% (query coverage 59%) and 91% (query coverage 21%) similarity with two previously described *L. rhamnosus* bacteriophages, Lrm1 (EU246945.1) and Lc-Nu (AY131267.2), respectively [36, 37]. However, nearly identical prophage sequences were detected in the genomes of *L. rhamnosus* CLS17 (NZ_JYCS01000023.1), *L. rhamnosus* B1 (NZ_NXEU01000011.1), and *L. rhamnosus* ASCC 3029 (NZ_MLJZ01000021.1). In our previous study, we described the release of phage particles by *L. rhamnosus* Pen [38]. Although the physiological role of continuous phage particle release in *Lactobacillus* is not evident, it may be beneficial for the bacterial host. It was previously suggested that such behaviour may enhance biofilm formation and promote horizontal gene transfer. On the other hand, by facilitating binding to human platelets, spontaneous prophage induction may also play an important role in bacterial virulence [39, 40]. Additionally, considering that such bacteriophages may be simultaneously released to the culture medium and that this phenomenon does not lead to complete lysis of the culture, microorganisms containing such phages may have high potential for application as safe food-grade vectors for presenting or producing various biological factors such as antigens, receptors, or virulence proteins [38, 41].

In conclusion, genomic analysis has confirmed the probiotic properties of *L. rhamnosus* Pen and may indicate new biotechnological applications of this industrially important strain. However, to understand the nature of the relationship between this probiotic bacterium and its phage, further studies for molecular and physiological characterisation of the released bacteriophage should be performed. We hope that future studies may further our knowledge of phage biology and shed new light on interactions between phages and bacteria.

## Additional files

**Additional file 1: Figure S1.** Transmission electron microscope micrograph of *Lactobacillus rhamnosus* strain Pen. Bacteria were stained negatively with 1% (w/v) phosphotungstic acid visualized with an LEO 912AB electron microscope. Scale bar indicates 1 µm (A) and 0.2 µm (B), respectively.

**Additional file 2: Figure S2.** Phylogenetic tree based on 16S rRNA encoding gene sequences for *Lactobacillus rhamnosus* Pen and selected strains belonging to the *Lactobacillus* genus. The three was constructed using the neighbour-joining method from 1000 bootstrapping replicates with the software package MEGA version 6.0.

**Additional file 3: Figure S3.** *Lactobacillus rhamnosus* Pen genome visualization showing coding sequence, COG categories, GC skew, GC content, rRNA and tRNA.

**Additional file 4: Table S1.** List of proteins involving with probiotic activity of *Lactobacillus rhamnosus* Pen.

## Abbreviations

ORF: open reading frame
COG: cluster of orthologous groups
CRISPR: clustered regularly interspaced short palindromic repeats
