# Supplementary figures and images for: Complete genome sequence of Lactobacillus rhamnosus Pen, a probiotic component of a medicine used in prevention of antibiotic-associated diarrhoea in children
Source: Gut Pathog. 2018 Feb 22;10:5. doi: 10.1186/s13099-018-0235-z (PMC5822663; doi:10.1186/s13099-018-0235-z)

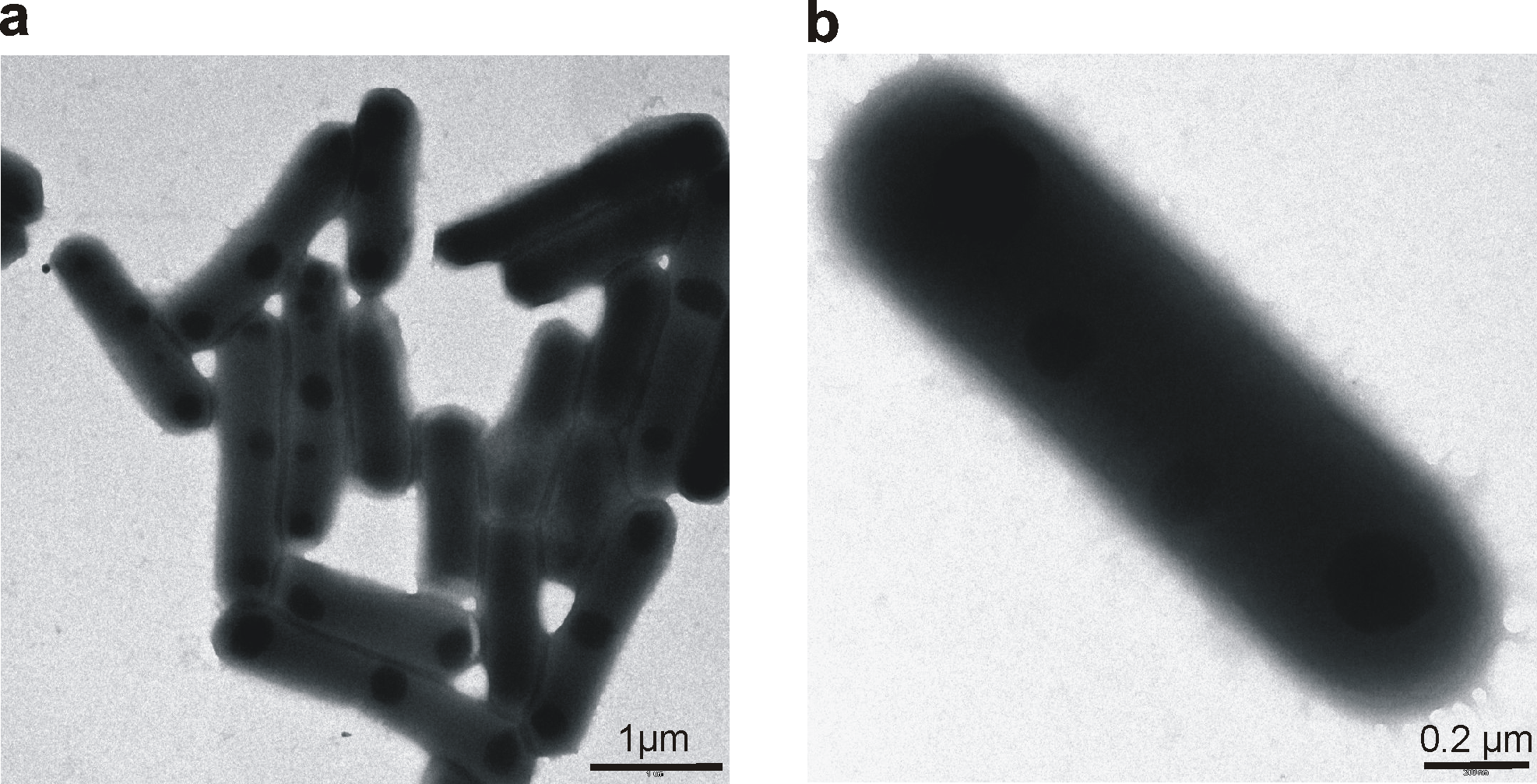

Supplement: Supplementary file 1 — Additional file 1: Figure S1. Transmission electron microscope micrograph of Lactobacillus rhamnosus strain Pen. Bacteria were stained negatively with 1% (w/v) phosphotungstic acid visualized with an LEO 912AB electron microscope. Scale bar indicates 1 µm (A) and 0.2 µm (B), respectively. [file 13099_2018_235_MOESM1_ESM.tif]

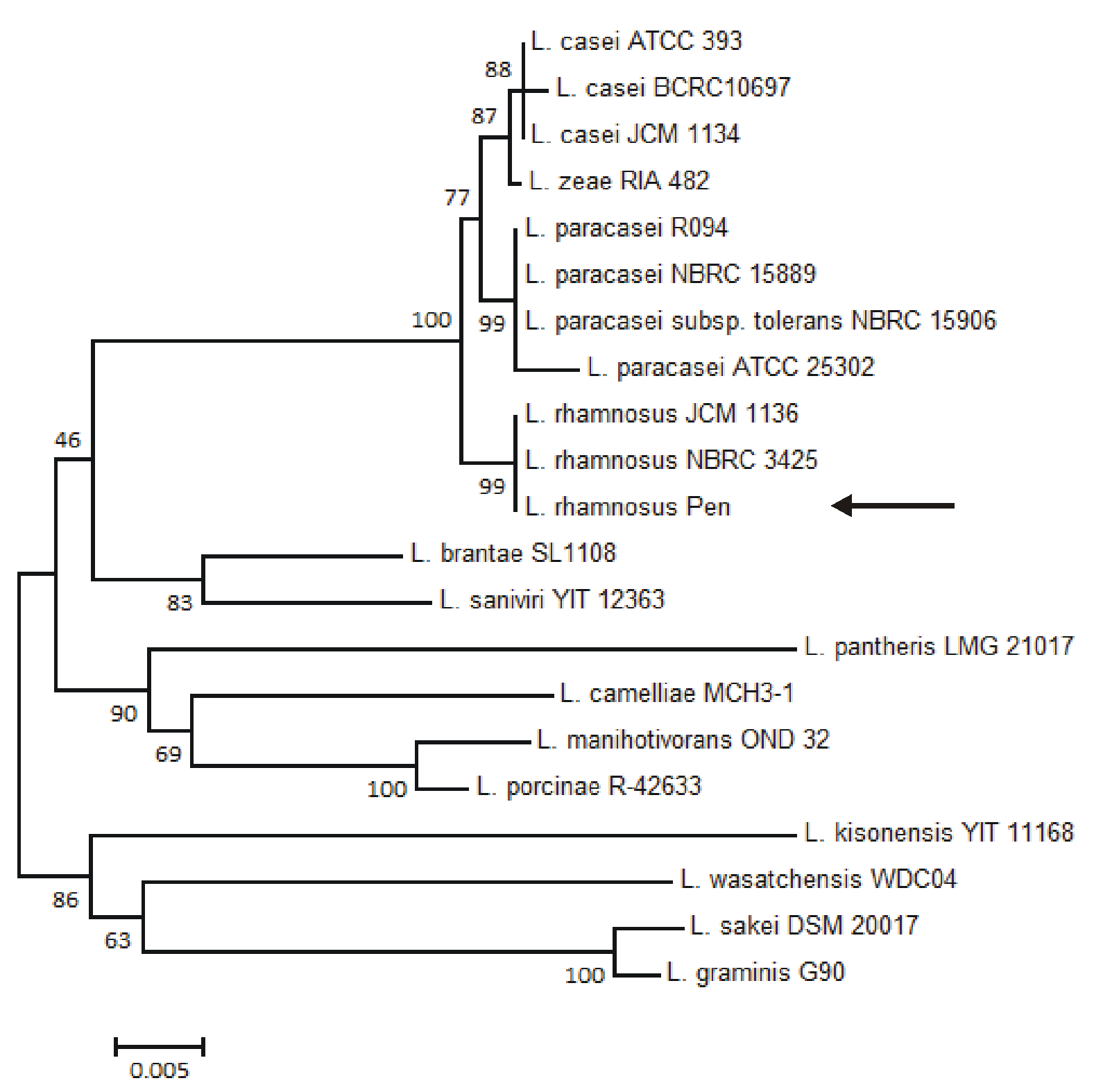

Supplement: Supplementary file 2 — Additional file 2: Figure S2. Phylogenetic tree based on 16S rRNA encoding gene sequences for Lactobacillus rhamnosus Pen and selected strains belonging to the Lactobacillus genus. The three was constructed using the neighbour-joining method from 1000 bootstrapping replicates with the software package MEGA version 6.0. [file 13099_2018_235_MOESM2_ESM.tif]

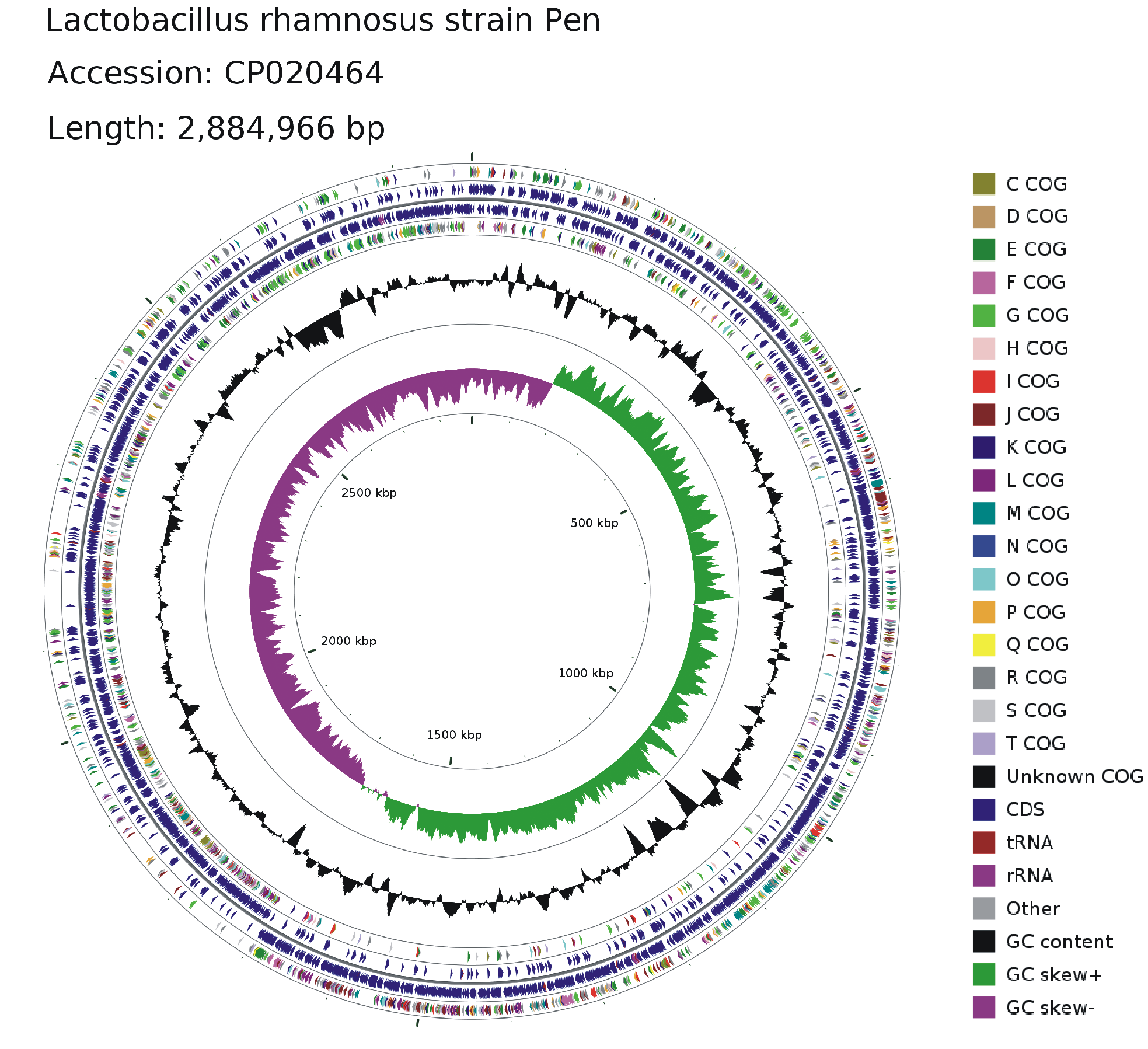

Supplement: Supplementary file 3 — Additional file 3: Figure S3. Lactobacillus rhamnosus Pen genome visualization showing coding sequence, COG categories, GC skew, GC content, rRNA and tRNA. [file 13099_2018_235_MOESM3_ESM.tif]
