# Supplementary material for: Complete genome sequence of Lactobacillus rhamnosus Pen, a probiotic component of a medicine used in prevention of antibiotic-associated diarrhoea in children
Source: Gut Pathog. 2018 Feb 22;10:5. doi: 10.1186/s13099-018-0235-z (PMC5822663; doi:10.1186/s13099-018-0235-z)
Supplement: Supplementary file 4 — Additional file 4: Table S1. List of proteins involving with probiotic activity of Lactobacillus rhamnosus Pen. [file 13099_2018_235_MOESM4_ESM.docx]

**Additional file 4**

**Table S1**

List of proteins involving with probiotic activity of *Lactobacillus rhamnosus* Pen.

| **Putative function** | **Protein number or genome location** |
| --- | --- |
| Adhesion | ARD32383.1, ARD32284.1, ARD32282.1, ARD32281.1, ARD32125.1, ARD31957.1, ARD31571.1, ARD31570.1, ARD31569.1, ARD31074.1, ARD33173.1, ARD32442.1, ARD32424.1, ARD32283.1, ARD32128.1, ARD32126.1, ARD33564.1, ARD31666.1, ARD33540.1, ARD31612.1, ARD31220.1, ARD31127.1, ARD31015.1, ARD33501.1, ARD33175.1, ARD33174.1, ARD33172.1, ARD32763.1, ARD31207.1, ARD33503.1, ARD33502.1 |
| Exopolysaccharide production | EPS biosynthesis cluster I;  CP020464.1: 1,071,496 - 1,089,524 (ARD31868.1 – ARD31883, ARD33555.1),  EPS biosynthesis cluster II;  CP020464.1: 1124159 – 144270 (ARD31912.1 - ARD31925.1) |
| Bacteriocin production | ARD31560.1, ARD31554.1, ARD31553.1, ARD31546.1, ARD31545.1, ARD31540.1, ARD31538.1, ARD31537.1, ARD31536.1, ARD31535.1 |
